# Behaviour Change Techniques in Computerized Cognitive Training for Cognitively Healthy Older Adults: A Systematic Review

**DOI:** 10.1007/s11065-022-09537-4

**Published:** 2022-02-14

**Authors:** Geeske Peeters, Irene L. Black, Sjaan R. Gomersall, Juliette Fritschi, Aoife Sweeney, Yasmin Guedes de Oliveira, Rogerio Panizzutti, Claire T. McEvoy, Amit Lampit

**Affiliations:** 1 Global Brain Health Institute, Trinity College Dublin, Dublin, Ireland; 2 Department of Geriatric Medicine, Radboud Institute of Health Science, Radboud University Medical Centre, Nijmegen, Netherlands; 3 Department of Clinical Nutrition and Dietetics, CHI Crumlin, Dublin, Ireland; 4 UCD Institute of Food and Health, University College Dublin, Dublin, Ireland; 5 School of Health and Rehabilitation Sciences, The University of Queensland, Brisbane, Australia; 6 School of Human Movement and Nutrition Sciences, The University of Queensland, Brisbane, Australia; 7 The Movement Mentor Physiotherapy, Brisbane, QLD Australia; 8 Centre for Public Health, Queen’s University Belfast, Belfast, Northern Ireland; 9 Institute of Psychiatry, Federal University of Rio de Janeiro, Rio de Janeiro, Brazil; 10 Department of Psychiatry, University of Melbourne, Melbourne, Australia; 11 Department of Neurology, Charité Universitätsmedizin Berlin, Berlin, Germany

**Keywords:** Behaviour change taxonomy, Brain training, Cognitive functioning, Adherence, Motivation

## Abstract

We aimed to describe behaviour change techniques (BCT) used in trials evaluating computerised cognitive training (CCT) in cognitively healthy older adults, and explore whether BCTs are associated with improved adherence and efficacy. The 90 papers included in a recent meta-analysis were reviewed for information about adherence and use of BCTs in accordance with the Behaviour Change Taxonomy. Studies using a specific BCT were compared with studies not using that BCT on efficacy (difference in Hedges’ g [Δg]) using three level meta-regression models and on median adherence using the Wilcoxon test. The median number of BCTs per study was 3 (interquartile range [IQR] = 2–5). ‘Feedback on behaviour’ (if provided by a person; Δg = -0.19, 95% confidence interval [CI] = -0.31;-0.07) and ‘non-specific reward’ (Δg = -0.19, CI = -0.34;-0.05) were associated with *lower* efficacy. Certain BCTs that involve personal contact may be beneficial, although none were statistically significantly associated with *greater* efficacy. The median percentage of adherence was 90% (IQR = 81–95). Adherence was higher in studies using the BCT ‘self-monitoring of behaviour’ and lower in studies using the BCT ‘graded tasks’ than studies not using these BCTs (p < 0.001). These findings provide first evidence that BCTs can influence both adherence to and efficacy of CCT programs in cognitively healthy older adults.

**Supplementary Information:**

The online version contains supplementary material available at 10.1007/s11065-022-09537-4.

## Introduction

Computerized cognitive training (CCT) is receiving increasing attention as a potential approach to prevent cognitive decline and dementia in older adults. Meta-analyses of published trials have indicated that CCT can improve cognitive functioning immediately in the post-training period in cognitively healthy older adults (Kelly et al., 2014; Lampit et al., 2014b). Evidence supporting the long-term benefit of cognitive training is limited as few studies have included long-term follow-up. Findings from the landmark ACTIVE trial suggest that the gains in cognitive function, particularly from training that focused on speed of processing, may translate into sustained maintenance of daily functioning, reduced risk of driving cessation and reduced incidence of dementia 10 years after training (Edwards et al., 2016, 2017; Rebok et al., 2014; Ross et al., 2016).

Lampit et al. (2014b, 2020) have published the two most comprehensive reviews to date of the effectiveness of CCT trials in cognitively healthy older adults (aged ≥ 60 years). Their first meta-analysis of 51 trials indicated a small but statistically significant effect on global cognitive functioning favouring CCT over the control group (Hedges’ g = 0.22, 95% confidence interval [CI] = 0.15–0.29) (Lampit et al., 2014b). An update of this review included a total of 90 papers and showed similar efficacy (Hedges’ g = 0.18, CI = 0.14–0.23) (Lampit et al., 2020). The majority of the trials included in these reviews were tightly controlled, group-based trials with relatively small numbers of participants (n < 50), and most employed high intensity protocols to deliver the CCT intervention, particularly in terms of the time commitment required for participants to complete the intervention (Lampit et al., 2014b, 2020).

To make CCT feasible to implement for a wide audience, translation of the evidence from these lab-based trials into large-scale community interventions is needed. This requires greater understanding about the precise nature and delivery of CCT intervention that can achieve maximum benefits for brain health. Motivation, engagement, and expectations are believed to influence the effectiveness of CCT (Boot et al., 2013; Foroughi et al., 2016). These factors can be influenced by behaviour change techniques (BCTs), which are the ‘active ingredients’ in an intervention designed to bring about the desired behaviour change (Michie et al., 2015). In the context of CCT, BCTs are strategies put in place to encourage adherence to the cognitive training protocol, namely, the frequency and time spent training. For examples of BCTs used in the context of CCT, please refer to the supplemental file. BCTs may influence efficacy of interventions *indirectly* via their influence on adherence. BCTs may also influence efficacy *directly* by enhancing participant’s engagement while training. Meta-analyses of lifestyle behaviour interventions have indicated that BCTs related to social support, goal setting, and self-monitoring are associated with improved adoption of diet and physical activity behaviours, while problem-solving techniques may be important for supporting sustained long-term behaviour change (Cradock et al., 2017; Lara et al., 2014; Olander et al., 2013).

To date, little attention has been given to the potential role of BCTs in the adoption and maintenance of cognitive training behaviours. It is unclear to what extent BCTs are being incorporated in CCT intervention design, which BCTs are used (if any), or whether there is any evidence supporting the usefulness of BCTs to improve the efficacy of CCT programs.

This review is the first to synthesise evidence from published trials about the use of behavioural strategies in CCT interventions. The aims of this review were to (a) describe BCTs used in CCT trials in cognitively healthy older adults, and (b) explore whether specific BCTs are associated with improved adherence and efficacy. The current review extends the recent review of 90 trials evaluating the efficacy of CCT in older adults without cognitive decline (Lampit et al., 2020) by conducting a comprehensive meta-analysis of BCTs involved these trials. The findings provide valuable information relevant to the design and implementation of future large-scale CCT interventions.

## Methods

This review was registered in PROSPERO (CRD42017071112) and was conducted in accordance with the PRIMSA guidelines. Note, however, that this review extends the previously published update by Lampit et al. (2020). Therefore, where relevant, we refer to that review to avoid unnecessary duplication.

### Search Strategy

The search was conducted in Medline, Embase, and PsycINFO and in August 2019. For the current review, all full texts were retrieved of the 90 studies included in the 2020 review that published results from randomised controlled trials of effects of CCT on one or more cognitive outcomes in healthy (i.e., no major cognitive, neurological, psychiatric, or sensory impairments) older adults (≥ 60 years) (Lampit et al., 2020). Studies were included if they involved CCT either as a single intervention or as part of a multidomain strategy, provided that the CCT intervention consisted of at least 50% of the total intervention load. The review did not exclude studies based on their delivery techniques or CCT content to allow a comparison of the efficacy of different approaches.

### Data Extraction and Coding of Behaviour Change Techniques

The following data were extracted from each of the 90 studies: (a) the instructed total duration to train, (b) the average time spent training, (c) the number of people who completed the study, (d) the number of people who dropped out, (e) reasons for drop out, (f) the number of people completing minimum required duration of training, (g) reasons for non-adherence, and (h) BCTs built into the intervention to support adoption or maintenance of new cognitive training behaviours. In addition, we used the published effect sizes (standard mean difference calculated as Hedges’ g) and variance for each of the studies (Lampit et al., 2020). The Hedges’ g values were used as a measure of efficacy and were calculated by pooling the results from all reported cognitive tests to reflect overall cognitive function (Lampit et al., 2020). For each study, data were extracted by a minimum of two investigators (GP, IB, SG, JF, CME, AS) and discrepancies were solved after discussion. If needed, a third investigator was consulted.

Coding of BCTs was based on the Behaviour Change Technique Taxonomy (BCTT v1) (Michie et al., 2015). This taxonomy describes 93 distinct BCTs divided into 16 clusters and has become the standard for classifying and reporting BCTs in the behaviour change literature. Only BCTs that targeted uptake of CCT behaviours in the active intervention group were coded. For four BCTs on monitoring and feedback, we specified whether the participants were monitored and whether feedback was given by a person or a computer. This distinction is not made in the BCTT, but we felt this mode of delivery could potentially modify the association between the BCT and the efficacy of CCT. For each paper, a minimum of two investigators (GP, IB, SG, JF, CME, AS) coded all papers on presence of BCTs. If needed, a third investigator was consulted to solve disagreements between the coders. All coders completed the BCTT training (https://www.bct-taxonomy.com/) and are certified BCT coders.

As generally few data were available on adherence, we had to adopt a pragmatic definition of adherence based on available information. Adherence was defined as either (in order of priority): [1] the percentage of average duration spent training relative to the instructed duration to train, [2] the percentage of participants in the study that met the studies’ criterion for adherence, or [3] the percentage of participants that completed the intervention.

### Risk of Bias and Study Quality

For a detailed description of the risk of bias in the trials, please refer to our previous publication (Lampit et al., 2020). Briefly, using the Risk of Bias 2.0 tool (Sterne et al., 2019) studies with high risk of bias or some concerns in the domains ‘bias due to missing outcome data’ or ‘bias in measurement of the outcome’ were considered as having a high risk of bias (n = 36) or some concerns (n = 29), respectively. As the published information on risk of bias and methodological quality was deemed sufficient, no additional quality scoring was done for the current review.

### Data Synthesis

First, the number and types of BCTs used across the 90 studies were described. Second, a three-level meta-regression (including the levels study, comparison, and outcome) was done including the BCT as a covariate and Hedges’ g as the outcome reflecting measure of efficacy of CCT over and above control. The models were repeated for each of the BCTs in the Taxonomy that were used in three or more studies. The regression coefficient reflects the difference in Hedges’ g between studies that did or did not use that BCT. Presented are the differences in Hedges’ g (Δg) and 95% confidence intervals (CI). Third, adherence rates and reasons for non-adherence were described. For each of the BCTs, median adherence rates were compared between studies that used or did not use that BCT using Wilcoxon rank sum test. The level of statistical significance was set at p < 0.05. Although, no cut-points or norms have been established for what constitutes a clinically meaningful difference in Hedges’ g values. We therefore applied the arbitrary cut-points of Δg < -0.10 and > 0.10, which is one standard deviation difference between subgroups and more than 50% difference in Hedge’s g relative to the overall efficacy across the 90 studies (g = 0.18).

## Results

Full texts were retrieved for all 90 studies. The 90 studies included a total of 117 subgroups and 1201 comparisons. The studies varied in sample size from 20 to 1398 and in mean age from 60.7 to 85.8 years (Table 1).

**Table 1:** Study characteristics

Author (year)	N	Mean age (years)	Training program	Adherence (%)^a^	N^o^ BCTs
Ackerman et al. (2010)	78	60.7	Wii Big brain Academy	-	5
Anguera et al. (2013)	46	66.3	NeuroRacer	94.1	9
Ball et al. (2002)	1398	73.6	Speed of processing	91.7	1
Ballesteros et al. (2014)	30	69.0	Lumosity	-	1
Ballesteros et al. (2017)	55	65.6	Lumosity	-	2
Barban et al. (2016)	114	70.9	SOCIABLE	-	1
Barban et al. (2017)	362	75.1	SOCIABLE	-	1
Barnes et al. (2013)	63	72.9	Posit Brain Fitness	76.2	2
Basak et al. (2008)	39	69.6	Rise of Nations	95.0	1
Belchior et al. (2013)	58	74	Medal of Honor, Tetris, UFOV Speed of Processing	-	7
Belchior et al. (2019)	54	73.2	Crazy Taxi, PS Insight	-	5
Berry et al. (2010)	30	71.9	PS Sweep Seeker	100	4
Boot et al. (2013b)	40 34	72.5 72.4	Brain Age 2 (Nintendo DS), Mario Cart DS	95.2 36.7	5 5
Bottiroli and Cavallini (2009)	44	66.2	Neuropsychological training software	-	2
Bozoki et al. (2013)	60	68.9	My better mind	90.6	9
Brehmer et al. (2012)	45	63.8	Cogmed	93.2	2
Buitenweg et al. (2017)	139	67.7	TAPASS	-	8
Burki et al. (2014)	65	68	In-house developed	-	2
Casutt et al. (2014)	46	72.8	In-house developed	88.5	2
Chan et al. (2015)	22	70.6	n-back tasks	-	3
Colzato et al. (2011)	60	67.6	In-house developed	66.7	1
Dahlin et al. (2008)	29	68.3	In-house developed	20.3	2
Desjardins-Crepeau et al. (2016)	76	72	Dual task training	-	2
Du et al. (2018)	31	69.5	Updating training	-	2
Dustman et al. (1992)	60	66.4	Atari video games	87.7	1
Edwards et al. (2002)	91	73.7	Speed of processing	93.2	4
Edwards et al. (2005)	126	75.6	Speed of processing	-	2
Edwards et al. (2015)	60	73.1	PS InSight		
Eggenberger et al. (2015)	47	79.7	In-house developed	79.8	2
Frankenmolen et al. (2018)	60	67.1	CogPack	-	3
Garcia-Campuzano et al. (2013)	24	76.7	In-house developed	-	2
Goghari and Lawlor-Savage (2017)	61	70.6	Brain Gymmer	85.0	5
Goldstein et al. (1997)	22	77.7	Tetris	100	4
Gronholm-Nyman et al. (2017)	33	68.5	In-house developed	-	4
Guye	142	70.4	Tatool	100	6
Hynes (2016)	25	71.0	In-house developed	100	3
Jaeggi et al. (2020)	155	72.9	n-back task	-	1
Ji et al. (2016)	34	70.1	In-house developed	-	4
Kuhn et al. (2017)	48	69.4	In-house developed	-	3
Lampit et al. (2014a)	77	72.1	COGPACK	79.5	1
Lange et al. (2015)	91	67.7	In-house developed	-	2
Lee et al. (2020)	59	69.7	Posit	82.8	3
Legault et al. (2011)	67	76	In-house developed	89.2	1
Li et al. (2010)	20	76.2	Dual-task training	90.9	1
Mahncke et al. (2006)	162	70.5	PS Brain Fitness	80.6	6
Maillot et al. (2012)	30	73.5	Exergames (Nintendo Wii)	93.8	4
McAvinue et al. (2013)	36	70.4	In-house developed	69.2	7
Millan-Calentie et al. (2015)	142	74.3	Telecognitio	100	1
Miller et al. (2013)	74	81.9	Dakim Brain Fitness	85.7	3
Mishra et al. (2014)	31	68.1	Distractor training	-	4
Nilsson et al. (2017)	123	69.6	In-house developed	-	2
Nouchi et al. (2012)	28	69.1	Brain Age (Nintendo DS)	87.5	3
Nouchi et al. (2016)	72	69.0	Speed of processing	-	2
Nouchi et al. (2019)	60	72.4	In-house developed	-	5
Nozawa et al. (2015)	23	68.0	In-house developed	-	5
O'Brien et al. (2013)	22	71.9	PS InSight	-	5
Payne et al. (2017)	40	67.9	ITrain	95.5	2
Peng et al. (2012)	78	69	Figure comparison	-	2
Pereira-Morales et al. (2017)	40	66	In-house developed	-	5
Peretz et al. (2011)	155	67.8	CogniFit	78.6	1
Pergher et al. (2018)	28	63.1	n-back task	-	4
Perrot et al. (2019)	46	65	Kawashima Brain Training, Super Mario Bros	100	3
Rasmusson et al. (1999)	46	78.4	CNT	92.9	3
Richmond et al. (2011)	40	66	In-house developed	87.0	3
Rolle et al. (2017)	40	68.7	Distributed attention task	-	6
Salminen et al. (2015)	36	64.9	Brain Twister	-	3
Sandberg et al. (2014)	30	69.3	In-house developed	93.8	1
Shatil (2013)	126	79.8	CogniFit	66.7	2
Shatil et al. (2014)	109	68	CogniFit	85.0	1
Simon et al. (2018a)	38	75.7	Cogmed	-	8
Simon et al. (2017)	39	70.7	Cogmed	-	8
Simpson et al. (2012)	31	62.3	MyBrainTrainer	97.1	4
Smith et al. (2009)	487	75.3	Posit Brain Fitness	92.1	6
Sosa et al. (2019)	35	74.7	Brain Age	90.9	2
Souders (2017)	60	72.4	Mind Frontiers	-	7
Stern (2011)	40	66.7	Space Fortress	85.0	3
ten Brinke et al. (2020)	79	72.1	Fit Brains	-	3
Toril et al. (2016)	39	71.6	Luminosity	95.0	4
van het Reve et al. (2014)	145	81.5	Cogniplus	88.0	1
van Muijden et al. (2012)	72	67.6	In-house developed	80.0	4
van Vleet et al. (2016a)	21	76.1	Tonic and Phasic Alertness Training	91.7	0
van Vleet et al. (2016b)	24	74.5	Tonic and Phasic Alertness Training	91.7	0
Vance et al. (2007)	159	75.1	Speed of processing	-	2
von Bastain et al. (2013)	57	68.4	WM training via Tatool	87.0	7
Wang et al. (2011)	52	64.2	In-house developed	86.7	5
Wayne et al. (2016)	26	65.0	Cogmed	100	5
Weicker et al. (2018)	60	67.8	WOME/RehaCom	85	3
West et al. (2020)	69	85.8	CogniFit	79.5	1
Wolinsky et al. (2011)	456	61.9	PS On the Road	91.1	6
Zimmerman et al. (2016)	67	61.2	Tatool	-	8

*M* mean, *SE* Standard Error, *SD* standard deviation, *I* intervention group, *C* control group, *BCT* behaviour change technique

^**a**^Adherence was calculated as the percentage of participants in the study that completed the intervention and/or met the studies’ criterion for adherence

The overall agreement in coding of BCTs between the two coders was high (99%), and the agreement in BCTs selected by at least one of the coders was 76%. Eighty-eight of the 90 studies reported using at least one BCT. The median number of BCTs used was 3 (interquartile range [IQR] = 2—5). Of the 93 BCTs in the taxonomy, 34 BCTs were identified in at least one study (Table 2). The most commonly used BCTs were ‘graded tasks’ (n = 62), ‘instruction on how to perform the behaviour’ (n = 51), ‘feedback on outcomes of behaviour’ (n = 29), and ‘adding objects to the environment ‘ (n = 19).

**Table 2:** Meta-analyses of the efficacy of computerized cognitive training interventions on cognitive function stratified by use of behaviour change techniques

**BEHAVIOUR CHANGE TECHNIQUE CLUSTER**	**N**^**o**^** studies using BCT**	**Difference in Hedge’s g between studies using and not using that BCT**
Behaviour change technique		Δg (CI)
**GOALS AND PLANNING**		
Goal setting (behaviour)	2	
Problem solving	0	
Goal setting (outcome)	2	
Action planning	4	0.02 (-0.37, 0.42)^a^
Review behaviour goals	1	
Discrepancy between current behaviour and goal	0	
Review outcome goals	1	
Behavioural contract	1	
Commitment	0	
**FEEDBACK AND MONITORING**		
Monitoring of behaviour by others without feedback	12	
By a person	9	-0.07 (-0.31, 0.16)
By a computer	2	-0.19 (-0.69, 0.32)^a^
Feedback on behaviour	12	
By a person	6	-0.19 (-0.31, -0.07)
By a computer	6	0.05 (-0.23, 0.34)
Self-monitoring of behaviour	8	-0.06 (-0.32, 0.21)
Self-monitoring of outcomes of behaviour	5	-0.04 (-0.83, 0.75)^a^
Monitoring of outcomes of behaviour without feedback	8	
By a person	6	0.10 (-0.20, 0.40)
By a computer	2	-0.22 (-0.66, 0.21)
Biofeedback	0	
Feedback on outcomes of behaviour	29	
By a person	6	0.14 (-0.31, 0.58)
By a computer	23	-0.09 (-0.18, 0.01)
**SOCIAL SUPPORT**		
Social support (unspecified)	8	-0.03 (-0.23, 0.17)
Social support (practical)	17	-0.09 (-0.20, 0.02)
Social support (emotional)	2	
**SHAPING KNOWLEDGE**		
Instruction on how to perform the behaviour	51	-0.05 (-0.14, 0.04)
Information about antecedents	0	
Re-attribution	0	
Behavioural experiments	0	
**NATURAL CONSEQUENCES**		
Information about health consequences	2	
Salience of consequences	0	
Information about social and environmental consequences	5	0.04 (-0.10, 0.17)
Monitoring of emotional consequences	0	
Anticipated regret	0	
Information about emotional consequences	0	
**COMPARISON OF BEHAVIOUR**		
Demonstration of the behaviour	4	-0.10 (-0.32, 0.13)
Social comparison	0	
Information about others approval	0	
**ASSOCIATIONS**		
Prompts/cues	5	-0.08 (-0.33, 0.16)
Cue signalling reward	0	
Reduce prompts/cues	0	
Remove access to the reward	0	
Remove aversive stimulus	0	
Satiation	0	
Exposure	0	
Associative learning	0	
**REPETITION AND SUBSTITUTION**		
Behavioural practice/rehearsal	15	-0.03 (-0.13, 0.07)
Behaviour substitution	0	
Habit formation	0	
Habit reversal	0	
Overcorrection	0	
Generalisation of target behaviour	0	
Graded tasks	62	-0.09 (-0.23, 0.05)
**COMPARISON OF OUTCOME**		
Credible source	4	0.25 (-0.09, 0.60) ^a^
Pros and cons	0	
Comparative imagining of future outcomes	0	
**REWARD AND THREAT**		
Material incentive (behaviour)	1	
Material reward (behaviour)	2	
Non-specific reward	5	-0.19 (-0.34, -0.05)
Social reward	1	
Social incentive	0	
Non-specific incentive	0	
Self-incentive	0	
Incentive (outcome)	0	
Self-reward	0	
Reward (outcome)	3	-0.04 (-0.45, 0.38)^1^
Future (punishment)	0	
**REGULATION**		
Pharmacological support	0	
Reduce negative emotions	0	
Conserving mental resources	0	
Paradoxical instructions	0	
**ANTECEDENT**		
Restructuring the physical environment	2	
Restructuring the social environment	1	
Avoidance/reducing exposure to cues for the behaviour	0	
Distraction	0	
Adding objects to the environment	19	-0.01 (-0.14, 0.11)
Body changes	0	
**IDENTITY**		
Identification of self as role model	0	
Framing/reframing	0	
Incompatible beliefs	0	
Valued self-identity	0	
Identity associated with changed behaviour	0	
**SCHEDULED CONSEQUENCES**		
Behaviour cost	0	
Punishment	0	
Remove reward	0	
Reward approximation	1	
Rewarding completion	1	
Situation-specific reward	0	
Reward incompatible behaviour	0	
Reward alternative behaviour	0	
Reduce reward frequency	0	
Remove punishment	0	
**SELF-BELIEF**		
Verbal persuasion about capability	3	-0.17 (-0.43, 0.10)^a^
Mental rehearsal of successful performance	0	
Focus on past success	0	
Self-talk	0	
**COVERT LEARNING**		
Imaginary punishment	0	
Imaginary reward	0	
Vicarious consequences	0	

Δg reflects the difference in Hedges’ g values between studies that did use a specific BCT and studies that did not use that BCT. A positive Δg reflects that the efficacy was higher in the studies that did use that specific BCT. A negative Δg reflects that the efficacy was lower in the studies that did use that specific BCT

*BCT* Behaviour Change Technique, CI 95% confidence interval

^a^Degrees of freedom were less than 4, resulting in wide confidence intervals; results must be interpreted with caution

**Fig. 1:**
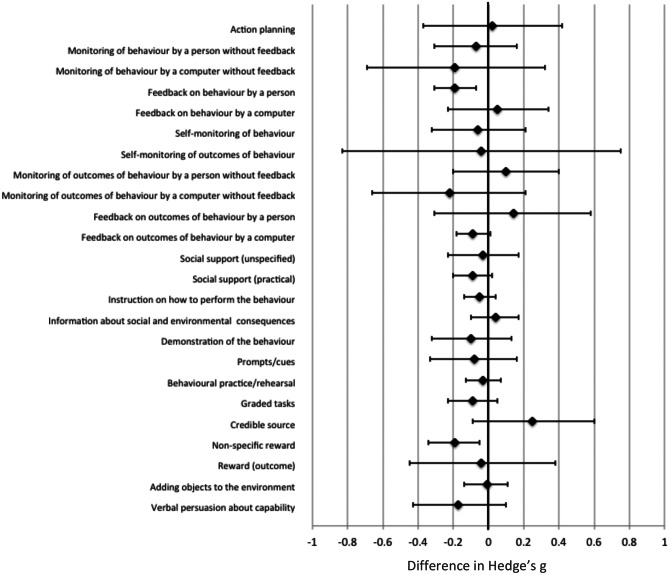
Meta-regression of computerized cognitive training interventions comparing the efficacy (expressed as Hedge’s g, x-axis) between studies that did and dit not use each of the behaviour change techniques (BCT, y-axis). The difference in Hedges’ g (Δg) reflects the difference in efficacy of the studies that did versus studies that did not include that BCT. A positive Δg reflects that the efficacy was higher in the studies that did use that specific BCT. A negative Δg reflects that the efficacy was lower in the studies that did use that specific BCT. Presented are the results for those BCTs that were used in at least three studies. Detailed results for all BCTs are presented in Table 2

We were able to reproduce the overall estimated effect size as reported by Lampit et al. (i.e., Hedges’ g = 0.18, CI = 0.14—0.23). No BCTs were associated with a statistically significant *greater* efficacy (Fig. 1; Table 2). However, Δg exceeded the cut-point for clinical relevance (> 0.10) for the BCTs ‘monitoring of outcomes of behaviour without feedback – by a person’, ‘feedback on outcomes of behaviour – by a person’ and ‘credible source’. The BCTs ‘feedback on behaviour – by a person’ (Δg = -0.19, CI = -0.31, -0.07) and ‘non-specific reward’ (Δg = -0.19, CI = -0.34, -0.05) were associated with *lower* efficacy (Fig. 1; Table 2). In addition, Δg exceeded the cut-point for clinical relevance (< -0.10) for the BCTs ‘monitoring of behaviour by others without feedback – by a computer’, ‘monitoring of outcomes of behaviour without feedback – by a computer’, ‘demonstration of the behaviour’ and ‘verbal persuasion about capability’. Supplemental file 1 lists examples for each of these BCTs.

Data on adherence were available for 52 studies (Table 1). Across these studies, the median adherence was high (90%, IQR = 81–95). Twenty-five studies reported the average time spent doing the cognitive training, which ranged from 75 to 100% of the total time participants were instructed to train. Forty-four studies reported the reasons for drop-out or non-adherence in the intervention group. The reported reasons for drop-out or non-adherence were excessive time commitment (eight papers), health problems (25 papers), lack of interest or motivation (11 papers), holiday or travel (five papers), lack of time (six papers), non-adherence (six papers), and disliking training (two papers) (Table 3). Studies that used the BCTs ‘self-monitoring of behaviour’, ‘monitoring of behaviour by others without feedback’ (p = 0.05) and ‘self-monitoring of outcomes of behaviour’ (p = 0.05) reported higher adherence rates than studies that did not use these BCTs (p < 0.001) (Table 4). Studies that used the BCT ‘graded tasks’ had lower adherence rates than studies that did not use that BCT (p < 0.001).

**Table 3:** Reasons for drop-out and non-adherence

Reason for drop-out or non-adherence	N^o^ papers	Papers that reported it (First Author, year)
Excessive time commitment	8	(Ball et al., 2002; Brehmer et al., 2012; Casutt et al., 2014; Edwards et al., 2015; Lampit et al., 2014a; Mahncke et al., 2006; Nilsson et al., 2017; van Muijden et al., 2012)
Health problems	25	(Ball et al., 2002; Brehmer et al., 2012; Edwards et al., 2015; Buschkuehl et al., 2008; von Bastian et al., 2013; Mayas et al., 2014; Miller et al., 2013; Nouchi et al., 2012; van Muijden et al., 2012; Ballesteros et al., 2017; Ballesteros et al., 2014; Buitenweg et al., 2017; Eggenberger et al., 2015; Goghari & Lawlor-Savage, 2017; Guye & von Bastian, 2017; Lange & Süß, 2015; Toril et al., 2016; van het Reve & de Bruin, 2014; Van Vleet et al., 2016; Desjardins-Crepeau et al., 2016; Nilsson et al., 2017; Frankenmolen et al., 2018; Simon et al., 2018; Weicker et al., 2018; West et al., 2020)
Lack of interest or motivation	11	(Ball et al., 2002; von Bastian et al., 2013; Miller et al., 2013; Peretz et al., 2011; Ballesteros et al., 2017; Buitenweg et al., 2017; Goghari & Lawlor-Savage, 2017; Guye & von Bastian, 2017; van het Reve & de Bruin, 2014; West et al., 2020; Sosa & Lagana, 2019)
Holiday/travel	5	(Ballesteros et al., 2014; Mayas et al., 2014; Miller et al., 2013; Simon et al., 2018; Van Vleet et al., 2016)
Lack of time	6	(Buitenweg et al., 2017; Guye & von Bastian, 2017; Lange & Süß, 2015; Nouchi et al., 2019; Simon et al., 2018; Sosa & Lagana, 2019)
Non-adherence	6	(Desjardins-Crepeau et al., 2016; Guye & von Bastian, 2017; Jaeggi et al., 2020; Mishra et al., 2014; Simpson et al., 2012; Smith et al., 2009)
Disliking training	2	(Buitenweg et al., 2017; Goghari & Lawlor-Savage, 2017)

**Table 4:** Comparing adherence in intervention groups between studies using and not using behaviour change techniques^a^

	BCT users		BCT non-users		
	N^o^ studies /N^o^ IG^b^	Median % [IQR]	N^o^ studies /N^o^ IG^b^	Median % [IQR]	p-value^c^
Monitoring of behaviour by others without feedback	5/7	95 [91–95]	42/48	88 [80–94]	0.05
Feedback on behaviour	4/5	95 [90–98]	43/50	89 [80–94]	0.07
Self-monitoring of behaviour	4/6	100 [97–100]	48/54	88 [80–93]	< 0.001
Self-monitoring of outcomes of behaviour	4/5	100 [89–100]	48/55	88 [80–94]	0.05
Feedback on outcomes of behaviour	15/16	92 [87–95]	32/39	88 [79–95]	0.28
Social support (unspecified)	7/7	91 [81–94]	45/53	89 [81–95]	0.70
Social support (practical)	8/8	92 [84–99]	44/52	89 [80–94]	0.29
Instruction on how to perform the behaviour	26/30	91 [84–95]	26/30	89 [79–94]	0.51
Information about social and environmental consequences	3/3	81 [69–93]	49/57	91 [84–95]	0.30
Prompts/cues	4/4	97 [87–100]	48/56	89 [80–94]	0.10
Behavioural practice/rehearsal	6/6	91 [90–92]	46/54	88 [80–95]	0.69
Graded tasks	32/37	85 [74–93]	20/23	94 [91–100]	< 0.001
Credible source	3/4	93 [92–94]	49/56	88 [80–95]	0.31
Non-specific reward	3/3	86 [85–91]	49/57	91 [80–95]	0.51
Reward (outcome)	3/3	92 [87–100]	49/57	89 [80–95]	0.34
Adding objects to the environment	9/11	92 [85–100]	43/49	88 [80–94]	0.21

*BCT* Behaviour Change Technique, IQR interquartile range

^**a**^Adherence was calculated as either (in order of priority): [1] the percentage of average duration spent training relative to the instructed duration to train, [2] the percentage of participants in the study that met the studies’ criterion for adherence, or [3] the percentage of participants that completed the intervention. The presented results are based on data from the 53 studies that reported on drop-out and/or adherence

^b^Number of studies and number of intervention groups (IG) with available data on adherence. This analysis was done only for those BCTs for which at least three studies that used that BCT had adherence data available

^c^The p-value was based on the Wilcoxon rank-sum test

## Discussion

### Main Findings

The presented results provide first evidence that specific BCTs can improve adherence to and negatively or positively affect efficacy of CCT programs in cognitively healthy older adults. Nearly all studies used at least one BCT. While 34 out of a potential 93 different BCTs were used across the studies, there was substantial variation in the BCTs that were used between studies. No BCTs were statistically significantly associated with positive effects on efficacy, while two BCTs were associated with negative effects on efficacy. Three BCTs were associated with positive effects on adherence and one with negative effects on adherence. As the average duration of the CCT interventions was 7.3 ± 4.2 (range 1.5–26) weeks, the results and discussion reflect the short-term adoption phase of CCT rather than the long-term maintenance (> 6 months) phase of CCT.

Few statistically significant differences in efficacy were found for any of the BCTs. This may either be explained by a true absence of associations or lack of statistical power. Moreover, many tests were done and Type 1 error cannot be ruled out, but it is yet unclear how best to account for multiple testing in meta-analyses (Bender et al., 2008). Also, a potential true absence of associations could be due to individual variation in preferences for BCTs, resulting no overall benefits on a group level. Although a large number of studies was included in the meta-regressions, some BCTs were used in only a small number of studies, resulting in wide confidence intervals. While these results need to be interpreted with caution, it is important to also consider the results in the context of clinical relevance. Seven BCTs exceed the threshold for clinical relevance (Δg of < -0.10 or > 0.10), suggesting that these BCTs may be interesting targets to explore further in future research to boost the efficacy of CCT interventions (Fig. 1, Table 2). These BCTs are discussed further below.

The contrasting directions of associations for the BCTs around feedback and monitoring may either reflect the lack of statistical power or suggest a complex interplay between what is monitored and how. Monitoring of the outcomes of behaviour (i.e., game performance) might be beneficial, while monitoring of the behaviour itself (i.e., time spent training) might be counter effective. Moreover, monitoring and feedback may translate to better efficacy if provided by a person but not if provided by a computer. Many CCT programs have an inbuilt option to track progress and time spent exercising. This feature is often used by researchers to monitor adherence to the training program. It is also used to send reminders to participants if adherence is dropping (i.e., prompting). Interestingly, ‘self-monitoring of the behaviour’, ‘feedback on behaviour’ ‘monitoring of the behaviour by others without feedback’ and (to a lesser extent) ‘prompts or cues’ appear to be associated with better adherence (Table 4), but not with better efficacy. This suggests that monitoring of behaviour and prompting may help to increase awareness of time spent training, but not necessarily be beneficial for improving the quality or intensity of training. While ‘feedback on outcomes of behaviour’ may be associated with better efficacy if the feedback was delivered by a person, ‘monitoring of behaviour by others without feedback’ by a computer tended to be associated with poorer efficacy (Fig. 1). Awareness of being monitored without receiving feedback may be perceived as stressful. Mixed methods research may provide insight into how the use of these BCTs influences experience, participation and engagement.

The positive direction of associations of ‘credible source’ and ‘feedback on performance by a person’ with efficacy suggest that personal contact is important. In contrast, there was a non-significant tendency toward lower efficacy in studies that provided verbal persuasion about capability (n = 3, Δg = -0.17, CI = -0.43, 0.10). Verbal persuasion involves telling the participant that they can successfully perform the training and arguing against self-doubts. Collectively, these findings suggest that personal contact is important, but that the type and form of information require further thought. Co-creation of CCT programs may be a good step forward to design programs that better align with the participants preferences and needs and avoid stress.

The BCT ‘non-specific rewards’ was associated with lower efficacy (Fig. 1, Table 2), but did not affect adherence (Table 4). If a reward is performed well, then the amount of effort participants invest is influenced by the expected reward. The non-specific nature of the reward may dilute the efficacy or may be de-motivating compared to a more specific, tangible award. Examples of the types of rewards that were offered included access to the game for three additional months post-intervention (Bozoki et al., 2013), a fun fact on the screen (Anguera et al., 2013), or points or animations based on their performance (Mayas et al., 2014). The lack of a positive association with adherence may be because these rewards were mostly linked to outcomes (i.e., performance during training) rather than behaviour (i.e., time spent training). It may be that the use of rewards alone, in absence of other BCTs, is not sufficient to stimulate adherence. It could also be that the type of reward is not meaningful to the participants. Note that some studies offered a financial reward for completion of the pre- and post-intervention assessments. As this reward was offered to improve adherence to the data collection, it was not coded as a BCT to improve adherence to the training program. The potential adverse effects of this BCT further emphasises the need to tailor the BCTs to the target population.

Three commonly used BCTs were ‘graded tasks’ (n = 62)’, ‘instruction on how to perform the behaviour’ (n = 51) and ‘adding objects to the environment ‘ (n = 19). One could argue that these three BCTs are essential to achieve any training effect. Participants need to receive instructions on the required behaviour to be able to adhere to the protocol (‘instruction on how to perform the behaviour’). The BCT ‘graded tasks’ was considered present if the program involved adaptation to changes in the participants level of cognitive function. The ability of the program to constantly adapt to the individual’s progress during training is fundamental to the efficacy of CCT (Lampit et al., 2014b). The BCT ‘adding objects to the environment’ was coded if studies provided software or hardware, or access to an online program. In accordance with the taxonomy manual (Michie et al., 2015), these three BCTs were coded only if the studies specifically described using these techniques in their papers. While not all studies described using these BCTs, it is likely that most, if not all, studies would have applied these BCTs in some form. This may explain why no beneficial effects are observed in adherence or efficacy when comparing studies coded as using these BCTs with those coded as not using these BCTs. Moreover, ‘graded tasks’ was associated with poorer adherence, which might be due to losing motivation when reaching a level that is perceived as too high. Detailed reporting of training protocols and consistent use of terminology would facilitate more accurate estimation of the effectiveness of these BCTs on adherence and efficacy.

Previous reviews of BCTs used in lifestyle interventions identified ‘goal setting (outcome)’ or ‘action planning’ as important (Cradock et al., 2017; Lara et al., 2014; Olander et al., 2013). In the current review, only four studies used ‘action planning’ (i.e., detailed planning of performance of the behaviour, e.g. frequency, duration, intensity), which had similar efficacy as the studies that did not use this BCT (Fig. 1). Two studies used ‘goal setting (outcome)’ (i.e., setting or agreeing on a goal defined in terms of the behaviour to be achieved). One of these studies had a markedly higher efficacy (Hedges’ g = 0.48, CI = 0.28–0.69) and the other study had a markedly lower efficacy (Hedges’ g = -0.24, CI = -0.55, 0.07) than the pooled efficacy across all studies (Hedges’ g = 0.18, CI = 0.14–0.23). More studies incorporating goal setting into CCT interventions are required for meaningful meta-analysis.

For some studies the definition of adherence is based on study completion, whereas for other studies the definition of adherence is based on time spent training. Drop-out during the intervention period and adherence to the protocol were generally poorly reported. The two definitions were combined to have sufficient number of papers for meaningful analyses. However, it is possible that BCTs affect drop-out differently than time spent training. Many studies present results from completers only. Some studies describe low adherence as a reason for exclusion from the analyses. This means that both the adherence and efficacy of CCT are likely overestimated. The high average adherence and narrow IQR (90%, IQR = 81–95) illustrate that there is little variation, which reduces the likelihood of finding statistically significant differences. A survey among 831 brain trainers identified participant characteristics associated with training time and performance. The survey found that being open to experience and having a positive attitude towards cognitively demanding situations were associated with longer training continuation (Double & Birney, 2016). In addition, being open to experience and believing intelligence to be modifiable were associated with better game performance (Double & Birney, 2016). Future research may explore how BCTs can tap into these personality traits and metacognitive beliefs to boost adherence and efficacy.

A surprising finding in the Lampit review was that interventions with lower frequency (1–3 session per week) had better efficacy than interventions with higher frequency (> 3 sessions per week) (Lampit et al., 2020). The current review of adherence and BCTs provides new insights that may explain this finding. First, all six studies with low frequency interventions were group-based interventions (Bottiroli & Cavallini, 2009; Peng et al., 2012; Rasmusson et al., 1999; Vance et al., 2007; Wolinsky et al., 2011) and four were supervised (Bottiroli & Cavallini, 2009; Vance et al., 2007; Wang et al., 2011; Wolinsky et al., 2011). Group-based interventions had better efficacy than home-based interventions. It may be that the apparent better outcomes of low frequency interventions are driven by the group-based, supervised nature of the interventions. This finding is consistent with other behavioural interventions (e.g., physical activity) and the challenge is to transition from supervised group settings to unsupervised individual training while maintaining adherence and efficacy. Second, four of the studies used a BCT that involved personal contact, including social support (Bottiroli & Cavallini, 2009; Wolinsky et al., 2011), credible source (Rasmusson et al., 1999; Wolinsky et al., 2011) and feedback on the outcomes of the behaviour (Wang et al., 2011). This may have boosted the efficacy of these studies. Third, interventions with lower frequency may be easier to fit in to the daily routine than interventions with higher frequency. Subsequently, adherence may be better in lower frequency interventions. However, the six studies provided little information on adherence and no strong conclusion can be drawn.

### Strengths and Limitations

Strengths of this review include the large number of studies and detailed information on efficacy (data were collected for all domains of cognitive function) and the BCTs were coded by a minimum of two trained coders with high agreement between coders. The following limitations need to be considered. First, this review included studies that predominantly involved unimodal CCT interventions. Multimodal interventions, typically a combination of CCT and exercise, were considered only if the CCT component consisted at least 50% of the full intervention. However, the increase in multimodal interventions indicates that researchers believe greater gains in cognitive function (or less decline) may be obtained from CCT in combination with wider risk factor management than from CCT alone. In multimodal interventions, the role of BCTs is even more crucial than in CCT only interventions as participants need to be encouraged to adhere to each of the intervention components. As use of BCTs is more integrated in lifestyle interventions than in CCT interventions, it is likely that use of BCTs are more common in multimodal interventions than in CCT only interventions. In a review of multimodal interventions, it may be challenging to tease out which BCTs were important for which intervention component. A critical motivation for this work is to inform design of future interventions. This review will be particularly relevant to inform which BCTs may be useful to promote CCT either as a single intervention or as part of a multimodal intervention. Further research is required to examine which BCTs are specifically effective in optimising adherence to CCT in single and multimodal interventions, and whether these differ from BCTs used in lifestyle interventions. Second, this review focused on BCTs used in trials evaluating the efficacy of CCT in healthy older adults. Hence, the current findings may not be generalizable to other types of cognitive training programs (e.g., paper-based cognitive training or multicomponent interventions) or other subgroups (e.g., older adults with mild cognitive impairment). Finally, the results were pooled across domains of cognitive function, creating an overall estimate of efficacy in terms of global cognitive function. Too few studies would be available for meaningful meta-analyses of the less-frequently used BCTs and of the less frequently measured cognitive domains. Moreover, there were no reasons to believe a priori that the influence of BCTs on efficacy may be different for domains of cognitive function, though future research would have to confirm this.

## Conclusion

The presented results provide first evidence that BCTs may influence both adherence to and efficacy of CCT programs in cognitively healthy older adults. The BCTs that appear to positively influence efficacy of CCT programs include ‘credible source’, ‘information about social and environmental consequences’ and ‘feedback on outcomes of behaviour’ (but only when the feedback is delivered by a person). The BCTs that appear to negatively influence efficacy include BCTs related to monitoring of and feedback on behaviours and outcomes of behaviour, particularly when provided by a computer. However, few associations were statistically significant and further research is required to verify the current findings. Moreover, future research should involve the target group in the design of CCT programs and application of BCTs to ensure the use of BCTs is aligned with their preferences and needs.

## Supplementary Information

Below is the link to the electronic supplementary material.

Supplementary file1 (DOCX 18 KB)

## Data Availability

No other data are available than published in this manuscript.

## Code Availability

Stata codes may be requested from the first author.

## References

[CR1] Ackerman, P. L., Kanfer, R., & Calderwood, C. (2010). Use it or lose it? Wii brain exercise practice and reading for domain knowledge. *Psychol Aging, 25*(4), 753–66. 10.1037/a0019277 10.1037/a0019277 PMC3000863 20822257

[CR2] Anguera JA, Boccanfuso J, Rintoul JL, Al-Hashimi O, Faraji F, Janowich J (2013). Video game training enhances cognitive control in older adults. Nature.

[CR3] Ball K, Berch DB, Helmers KF, Jobe JB, Leveck MD, Marsiske M (2002). Effects of cognitive training interventions with older adults: A randomized controlled trial. JAMA.

[CR4] Ballesteros S, Mayas J, Prieto A, Ruiz-Marquez E, Toril P, Reales JM (2017). Effects of Video Game Training on Measures of Selective Attention and Working Memory in Older Adults: Results from a Randomized Controlled Trial. Frontiers in Aging Neuroscience.

[CR5] Ballesteros S, Prieto A, Mayas J, Toril P, Pita C, Ponce de Leon L (2014). Brain training with non-action video games enhances aspects of cognition in older adults: A randomized controlled trial. Frontiers in Aging Neuroscience.

[CR6] Barban, F., Annicchiarico, R., Melideo, M., Federici, A., Lombardi, M. G., Giuli, S., et al. (2017). Reducing Fall Risk with Combined Motor and Cognitive Training in Elderly Fallers. *Brain Sciences, 7*(2), 19. 10.3390/brainsci7020019 10.3390/brainsci7020019 PMC5332962 28208604

[CR7] Barban, F., Annicchiarico, R., Pantelopoulos, S., Federici, A., Perri, R., Fadda, L., et al. (2016). Protecting cognition from aging and Alzheimer's disease: a computerized cognitive training combined with reminiscence therapy. *International Journal of Geriatric Psychiatry, 31*, 340–348. 10.1002/gps.4328 10.1002/gps.4328 26205305

[CR8] Barnes, D. E., Santos-Modesitt, W., Poelke, G., Kramer, A. F., Castro, C., Middleton, L. E., et al. (2013). The Mental Activity and eXercise (MAX) trial: a randomized controlled trial to enhance cognitive function in older adults. *JAMA internal medicine, 173*(9), 797–804. 10.1001/jamainternmed.2013.189 PMC5921904 23545598

[CR9] Basak, C., Boot, W. R., Voss, M. W., & Kramer, A. F. (2008). Can training in a real-time strategy video game attenuate cognitive decline in older adults? *Psychol Aging, 2*(4), 765–77. 10.1037/a0013494. PMID: 19140648; PMCID: PMC4041116. 10.1037/a0013494 PMC4041116 19140648

[CR10] Belchior, P., Marsiske, M., Sisco, S. M., Yam, A., Bavelier, D., Ball, K., et al. (2013). Video game training to improve selective visual attention in older adults. *Computers in human behavior, 29*(4), 1318–1324. 10.1016/j.chb.2013.01.034 PMC3758751 24003265

[CR11] Belchior, P., Yam, A., Thomas, K. R., Bavelier, D., Ball, K. K., Mann, W. C., et al. (2019). Computer and Videogame Interventions for Older Adults' Cognitive and Everyday Functioning. *Games Health J, 8*(2), 129–143. 10.1089/g4h.2017.0092 10.1089/g4h.2017.0092 PMC6482895 30273002

[CR12] Bender R, Bunce C, Clarke M, Gates S, Lange S, Pace NL (2008). Attention should be given to multiplicity issues in systematic reviews. Journal of Clinical Epidemiology.

[CR13] Berry, A. S., Zanto, T. P., Clapp, W. C., Hardy, J. L., Delahunt, P. B., Mahncke, H. W., et al. (2010). The influence of perceptual training on working memory in older adults. *PLoS One, 5*(7), e11537. 10.1371/journal.pone.0011537 10.1371/journal.pone.0011537 PMC2904363 20644719

[CR14] Boot, W. R., Champion, M., Blakely, D. P., Wright, T., Souders, D., & Charness, N. (2013b). Video games as a means to reduce age-related cognitive decline: attitudes, compliance, and effectiveness. *Frontiers in psychology, 4*, 31. 10.3389/fpsyg.2013.00031 PMC3561600 23378841

[CR15] Boot WR, Simons DJ, Stothart C, Stutts C (2013). The Pervasive Problem With Placebos in Psychology. Perspectives on Psychological Science.

[CR16] Bottiroli, S., & Cavallini, E. (2009). Can computer familiarity regulate the benefits of computer-based memory training in normal aging? A study with an Italian sample of older adults. *Neuropsychology, Development, and Cognition. Section B: Aging, Neuropsychology and Cognition, 16*(4), 401–418. 10.1080/13825580802691763. 10.1080/13825580802691763 19253069

[CR17] Bozoki A, Radovanovic M, Winn B, Heeter C, Anthony JC (2013). Effects of a computer-based cognitive exercise program on age-related cognitive decline. Archives of Gerontology and Geriatrics.

[CR18] Bürki, C. N., Ludwig, C., Chicherio, C., & de Ribaupierre, A. (2014). Individual differences in cognitive plasticity: an investigation of training curves in younger and older adults. *Psychological Research, 78*(6), 821–835. 10.1007/s00426-014-0559-3 24652343

[CR19] Brehmer Y, Westerberg H, Backman L (2012). Working-memory training in younger and older adults: Training gains, transfer, and maintenance. Frontiers in Human Neuroscience.

[CR20] Buitenweg JIV, van de Ven RM, Prinssen S, Murre JMJ, Ridderinkhof KR (2017). Cognitive Flexibility Training: A Large-Scale Multimodal Adaptive Active-Control Intervention Study in Healthy Older Adults. Frontiers in Human Neuroscience.

[CR21] Buschkuehl M, Jaeggi SM, Hutchison S, Perrig-Chiello P, Dapp C, Muller M (2008). Impact of working memory training on memory performance in old-old adults. Psychology and Aging.

[CR22] Casutt G, Theill N, Martin M, Keller M, Jancke L (2014). The drive-wise project: Driving simulator training increases real driving performance in healthy older drivers. Frontiers in Aging Neuroscience.

[CR23] Chan JS, Wu Q, Liang D, & Yan JH. (2015). Visuospatial working memory training facilitates visually-aided explicit sequence learning. Acta Psychol (Amst), 161, 145–53. 10.1016/j.actpsy.2015.09.008 10.1016/j.actpsy.2015.09.008 26398484

[CR24] Colzato, L. S., van Muijden, J., Band, G. P., & Hommel, B. (2013). Genetic Modulation of Training and Transfer in Older Adults: BDNF ValMet Polymorphism is Associated with Wider Useful Field of View. *Front Psychol, 2*, 199. 10.3389/fpsyg.2011.0019 10.3389/fpsyg.2011.00199 PMC3164110 21909331

[CR25] Cradock KA, ÓLaighin, G., Finucane, F. M., Gainforth, H. L., Quinlan, L. R., & Ginis, K. A.  (2017). Behaviour change techniques targeting both diet and physical activity in type 2 diabetes: A systematic review and meta-analysis. The International Journal of Behavioral Nutrition and Physical Activity.

[CR26] Dahlin, E., Nyberg, L., Bäckman, L., & Neely, A. S. (2008). Plasticity of executive functioning in young and older adults: immediate training gains, transfer, and long-term maintenance. *Psychol Aging, 23*(4), 720–30. 10.1037/a0014296 10.1037/a0014296 19140643

[CR27] Desjardins-Crepeau L, Berryman N, Fraser SA, Vu TT, Kergoat MJ, Li KZ (2016). Effects of combined physical and cognitive training on fitness and neuropsychological outcomes in healthy older adults. Clinical Interventions in Aging.

[CR28] Double KS, Birney DP (2016). The effects of personality and metacognitive beliefs on cognitive training adherence and performance. Personality and Individual Differences.

[CR29] Du, X., Ji, Y., Chen, T., Tang, Y., & Han, B. (2018). Can working memory capacity be expanded by boosting working memory updating efficiency in older adults?. *Psychology and Aging, 33*(8), 1134. 10.1037/pag0000311 30475011

[CR30] Dustman, R. E., Emmerson, R. Y., Steinhaus, L. A., Shearer, D. E., & Dustman, T. J. (1992). The effects of videogame playing on neuropsychological performance of elderly individuals. *Journal of Gerontol, 47*(3), 168–71. 10.1093/geronj/47.3.p168 10.1093/geronj/47.3.p168 1573200

[CR31] Edwards, J., Wadley, V., Myers, R. E., Roenker, D. L., Cissell, G., & Ball, K., (2002). Transfer of a speed of processing intervention to near and far cognitive functions. *Gerontology, 48*(5), 329–40. 10.1159/000065259 10.1159/000065259 12169801

[CR32] Edwards, J. D., Wadley, V. G., Vance, D. E., Wood, K., Roenker, D. L., & Ball, K. K. (2005). The impact of speed of processing training on cognitive and everyday performance. *Aging & Mental Health. 9*(3), 262–71. 10.1080/13607860412331336788 10.1080/13607860412331336788 16019280

[CR33] Edwards, J. D., Valdes, E. G., Peronto, C., Castora-Binkley, M., Alwerdt, J., Andel, R., et al. (2015). The Efficacy of InSight Cognitive Training to Improve Useful Field of View Performance: A Brief Report. *Journals of Gerontology. Series B: Psychological Sciences and Social Sciences, 70*(3), 417–422. 10.1093/geronb/gbt113 24211819

[CR34] Edwards JD, Xu H, Clark D, Ross LA, Unverzagt FW (2016). The active study: What we have learned and what is next? cognitive training reduces incident dementia across ten years. Alzheimer's & Dementia.

[CR35] Edwards JD, Xu H, Clark DO, Guey LT, Ross LA, Unverzagt FW (2017). Speed of processing training results in lower risk of dementia. Alzheimer's & Dementia.

[CR36] Eggenberger P, Schumacher V, Angst M, Theill N, de Bruin ED (2015). Does multicomponent physical exercise with simultaneous cognitive training boost cognitive performance in older adults? A 6-month randomized controlled trial with a 1-year follow-up. Clinical Interventions in Aging.

[CR37] Foroughi CK, Monfort SS, Paczynski M, McKnight PE, Greenwood PM (2016). Placebo effects in cognitive training. Proceedings of the National Academy of Sciences of the United States of America.

[CR38] Frankenmolen NL, Overdorp EJ, Fasotti L, Claassen J, Kessels RPC, Oosterman JM (2018). Memory Strategy Training in Older Adults with Subjective Memory Complaints: A Randomized Controlled Trial. Journal of the International Neuropsychological Society.

[CR39] Garcia‐Campuzano, M. T., Virues‐Ortega, J., Smith, S., & Moussavi, Z. (2013). Effect of cognitive training targeting associative memory in the elderly: a small randomized trial and a longitudinal evaluation. 10.1111/jgs.12574 24329837

[CR40] Goghari VM, Lawlor-Savage L (2017). Comparison of Cognitive Change after Working Memory Training and Logic and Planning Training in Healthy Older Adults. Frontiers in Aging Neuroscience.

[CR41] Goldstein, J., Cajko, L., Oosterbroek, M., Michielsen, M., Van Houten, O., & Salverda, F. (1997). Video games and the elderly. *Social Behavior and Personality: an international journal, 25*(4), 345–352.

[CR42] Grönholm-Nyman, P,. Soveri, A., Rinne, J. O., Ek, E., Nyholm, A., Stigsdotter Neely, A., et al. (2017). Limited Effects of Set Shifting Training in Healthy Older Adults. I, 69. 10.3389/fnagi.2017.00069 10.3389/fnagi.2017.00069 PMC5362725 28386226

[CR43] Guye S, von Bastian CC (2017). Working memory training in older adults: Bayesian evidence supporting the absence of transfer. Psychology and Aging.

[CR44] Hynes, S.M. (2016). Internet, home-based cognitive and strategy training with older adults: a study to assess gains to daily life. Aging clinical and experimental research, 28(5), 1003–1008. 10.1007/s40520-015-0496-z 10.1007/s40520-015-0496-z 26589906

[CR45] Jaeggi, S. M., Buschkuehl, M., Parlett-Pelleriti, C. M., Moon, S. M., Evans, M., Kritzmacher, A., et al. (2020). Investigating the Effects of Spacing on Working Memory Training Outcome: A Randomized, Controlled, Multisite Trial in Older Adults. *Journals of Gerontology. Series B: Psychological Sciences and Social Sciences, 75*(6), 1181–1192. 10.1093/geronb/gbz090 PMC7265810 31353413

[CR46] Ji, Y., Wang, J., Chen, T., Du, X., & Zhan, Y. (2016). Plasticity of inhibitory processes and associated far-transfer effects in older adults. *Psychology and Aging, 31*(5), 415–429. 10.1037/pag0000102 10.1037/pag0000102 27243762

[CR47] Kelly ME, Loughrey D, Lawlor BA, Robertson IH, Walsh C, Brennan S (2014). The impact of cognitive training and mental stimulation on cognitive and everyday functioning of healthy older adults: A systematic review and meta-analysis. Ageing Research Reviews.

[CR48] Kühn, S., Lorenz, R. C., Weichenberger, M., Becker, M., Haesner, M., et al. (2017). Taking control! Structural and behavioural plasticity in response to game-based inhibition training in older adults. *Neuroimage, 156*, 199–206. 10.1016/j.neuroimage.2017.05.026 10.1016/j.neuroimage.2017.05.026 28527788

[CR49] Lampit A, Hallock H, Moss R, Kwok S, Rosser M, Lukjanenko M (2014). The timecourse of global cognitive gains from supervised computer-assisted cognitive training: A randomised, active-controlled trial in elderly with multiple dementia risk factors. J Prev Alz Dis.

[CR50] Lampit A, Hallock H, Valenzuela M (2014). Computerized cognitive training in cognitively healthy older adults: A systematic review and meta-analysis of effect modifiers. PLoS Medicine.

[CR51] Lampit, A., Malmberg Gavelin, H., Sabates, J., Launder, N. H., Hallock, H., Finke, C., et al. (2020). Computerized Cognitive Training in Cognitively Healthy Older Adults: A Systematic Review and Network Meta-Analysis. *medRxiv*, 2020.2010.2007.20208306. 10.1101/2020.10.07.20208306

[CR52] Lange S, Süß H-M (2015). Experimental Evaluation of Near- and Far-Transfer Effects of an Adaptive Multicomponent Working Memory Training. Applied Cognitive Psychology.

[CR53] Lara J, Evans EH, O’Brien N, Moynihan PJ, Meyer TD, Adamson AJ (2014). Association of behaviour change techniques with effectiveness of dietary interventions among adults of retirement age: A systematic review and meta-analysis of randomised controlled trials. BMC Medicine.

[CR54] Lee, H. K., Kent, J. D., Wendel, C., Wolinsky, F. D., Foster, E. D., Merzenich, M. M., et al. (2020). Home-Based, Adaptive Cognitive Training for Cognitively Normal Older adults: Initial Efficacy Trial. *The Journals of Gerontology: Series B, 75*(6), 1144–1154. 10.1093/geronb/gbz073 10.1093/geronb/gbz073 PMC7265807 31140569

[CR55] Legault, C., Jennings, J. M., Katula, J. A., Dagenbach, D., Gaussoin, S. A., Sink, K. M. et al. (2011). SHARP-P Study Group. Designing clinical trials for assessing the effects of cognitive training and physical activity interventions on cognitive outcomes: the Seniors Health and Activity Research Program Pilot (SHARP-P) study, a randomized controlled trial. *BMC Geriatrics, 11*, 27. 10.1186/1471-2318-11-27 10.1186/1471-2318-11-27 PMC3126708 21615936

[CR56] Li, K. Z., Roudaia, E., Lussier, M., Bherer, L., Leroux, A., & McKinley, P. A. (2010). Benefits of cognitive dual-task training on balance performance in healthy older adults. *Journals of Gerontology Series A: Biomedical Sciences and Medical Sciences, 65*(12), 1344–1352. 10.1093/gerona/glq151 10.1093/gerona/glq151 20837662

[CR57] Mahncke HW, Connor BB, Appelman J, Ahsanuddin ON, Hardy JL, Wood RA (2006). Memory enhancement in healthy older adults using a brain plasticity-based training program: A randomized, controlled study. Proceedings of the National Academy of Sciences of the United States of America.

[CR58] Maillot, P., Perrot, A., & Hartley, A. (2012). Effects of interactive physical-activity video-game training on physical and cognitive function in older adults. Psychology and Aging, 27(3), 589–600. 10.1037/a0026268 10.1037/a0026268 22122605

[CR59] Mayas J, Parmentier FB, Andres P, Ballesteros S (2014). Plasticity of attentional functions in older adults after non-action video game training: A randomized controlled trial. PLoS ONE.

[CR60] McAvinue, L. P., Golemme, M., Castorina, M., Tatti, E., Pigni, F. M., & Salomone, S. (2013). An evaluation of a working memory training scheme in older adults. *Front Aging Neurosci, 23*(5), 20. 10.3389/fnagi.2013.00020 10.3389/fnagi.2013.00020 PMC3662021 23734126

[CR61] Michie S, Wood CE, Johnston M, Abraham C, Francis JJ, Hardeman W (2015). Behaviour change techniques: The development and evaluation of a taxonomic method for reporting and describing behaviour change interventions (a suite of five studies involving consensus methods, randomised controlled trials and analysis of qualitative data). Health Technology Assessment.

[CR62] Millán-Calenti, J. C., Lorenzo, T., Núñez-Naveira, L., Buján, A., Rodríguez-Villamil, J. L., & Maseda, A. (2015). Efficacy of a computerized cognitive training application on cognition and depressive symptomatology in a group of healthy older adults: A randomized controlled trial. *Archives of Gerontology and Geriatrics, 61*(3):337–43. 10.1016/j.archger.2015.08.015 10.1016/j.archger.2015.08.015 26321734

[CR63] Miller KJ, Dye RV, Kim J, Jennings JL, O'Toole E, Wong J (2013). Effect of a computerized brain exercise program on cognitive performance in older adults. American Journal of Geriatric Psychiatry.

[CR64] Mishra J, de Villers-Sidani E, Merzenich M, Gazzaley A (2014). Adaptive training diminishes distractibility in aging across species. Neuron.

[CR65] Nilsson J, Lebedev AV, Rydstrom A, Lovden M (2017). Direct-Current Stimulation Does Little to Improve the Outcome of Working Memory Training in Older Adults. Psychological Science.

[CR66] Nouchi R, Kobayashi A, Nouchi H, Kawashima R (2019). Newly Developed TV-Based Cognitive Training Games Improve Car Driving Skills, Cognitive Functions, and Mood in Healthy Older Adults: Evidence From a Randomized Controlled Trial. Frontiers in Aging Neuroscience.

[CR67] Nouchi, R., Saito, T., Nouchi, H., & Kawashima, R. (2016). Small Acute Benefits of 4 Weeks Processing Speed Training Games on Processing Speed and Inhibition Performance and Depressive Mood in the Healthy Elderly People: Evidence from a Randomized Control Trial. *Frontiers in aging neuroscience, 23*(8), 302. 10.3389/fnagi.2016.00302 10.3389/fnagi.2016.00302 PMC5179514 28066229

[CR68] Nouchi R, Taki Y, Takeuchi H, Hashizume H, Akitsuki Y, Shigemune Y (2012). Brain training game improves executive functions and processing speed in the elderly: A randomized controlled trial. PLoS ONE.

[CR69] Nozawa, T., Taki, Y., Kanno, A., Akimoto, Y., Ihara, M., Yokoyama, R., et al. (2015). Effects of Different Types of Cognitive Training on Cognitive Function, Brain Structure, and Driving Safety in Senior Daily Drivers: A Pilot Study. *Behavioral Neurology, 2015*, 525901. 10.1155/2015/525901 10.1155/2015/525901 PMC4487932 26161000

[CR70] O'Brien, J. L., Edwards, J. D., Maxfield, N. D., Peronto, C. L., Williams, V. A., & Lister, J. J. (2013). Cognitive training and selective attention in the aging brain: an electrophysiological study. *Clinical neurophysiology, 124*(11), 2198–208. 10.1016/j.clinph.2013.05.012 10.1016/j.clinph.2013.05.012 23770088

[CR71] Olander EK, Fletcher H, Williams S, Atkinson L, Turner A, French DP (2013). What are the most effective techniques in changing obese individuals' physical activity self-efficacy and behaviour: A systematic review and meta-analysis. The International Journal of Behavioral Nutrition and Physical Activity.

[CR72] Payne, B. R., & Stine-Morrow, E. A. L. (2017). The Effects of Home-Based Cognitive Training on Verbal Working Memory and Language Comprehension in Older Adulthood. *Frontiers in Aging Neuroscience, 8*(9), 256. 10.3389/fnagi.2017.00256 10.3389/fnagi.2017.00256 PMC5550674 28848421

[CR73] Peng H, Wen J, Wang D, Gao Y (2012). The Impact of Processing Speed Training on Working Memory in Old Adults. Journal of Adult Development.

[CR74] Pereira-Morales, A. J., Cruz-Salinas, A. F., Aponte, J., & Pereira-Manrique, F. (2018). Efficacy of a computer-based cognitive training program in older people with subjective memory complaints: a randomized study. *International Journal of Neuroscience, 128*(1), 1–9. 10.1080/00207454.2017.1308930 10.1080/00207454.2017.1308930 28316267

[CR75] Peretz C, Korczyn AD, Shatil E, Aharonson V, Birnboim S, Giladi N (2011). Computer-based, personalized cognitive training versus classical computer games: A randomized double-blind prospective trial of cognitive stimulation. Neuroepidemiology.

[CR76] Pergher, V., Wittevrongel, B., Tournoy, J., Schoenmakers, B., & Van Hulle, M. M. (2018). N-back training and transfer effects revealed by behavioral responses and EEG. *Brain and Behavior, 8*(11), e01136. 10.1002/brb3.1136 10.1002/brb3.1136 PMC6236237 30350357

[CR77] Perrot, A., Maillot, P., & Hartley, A. (2019). Cognitive Training Game Versus Action Videogame: Effects on Cognitive Functions in Older Adults. *Games Health Journal, 8*(1), 35–40. 10.1089/g4h.2018.0010 10.1089/g4h.2018.0010 30376364

[CR78] Rasmusson DX, Rebok GW, Bylsma FW, Brandt J (1999). Effects of Three Types of Memory Training in Normal Elderly. Aging, Neuropsychology, and Cognition.

[CR79] Rebok GW, Ball K, Guey LT, Jones RN, Kim HY, King JW (2014). Ten-year effects of the advanced cognitive training for independent and vital elderly cognitive training trial on cognition and everyday functioning in older adults. Journal of the American Geriatrics Society.

[CR80] Richmond, L. L, Morrison, A. B., Chein, J. M., & Olson, I. R. (2011). Working memory training and transfer in older adults. *Psychology and Aging, 26*(4), 813–22. 10.1037/a0023631 10.1037/a0023631 21707176

[CR81] Rolle, C. E., Anguera, J. A., Skinner, S. N., Voytek, B., Gazzaley, A. (2017). Enhancing Spatial Attention and Working Memory in Younger and Older Adults. *Journal of cognitive neuroscience, 29*(9), 1483–1497. 10.1162/jocn_a_01159 10.1162/jocn_a_01159 PMC5903566 28654361

[CR82] Ross LA, Freed SA, Edwards JD, Phillips CB, Ball K (2016). The Impact of Three Cognitive Training Programs on Driving Cessation Across 10 Years: A Randomized Controlled Trial. The Gerontologist.

[CR83] Salminen, T., Frensch, P., Strobach, T., & Schubert, T. (2016). Age-specific differences of dual n-back training. *Aging, Neuropsychology, and Cognition, 23*(1), 18–39. 10.1080/13825585.2015.1031723 10.1080/13825585.2015.1031723 25867501

[CR84] Sandberg, P., Rönnlund, M., Nyberg, L., & Stigsdotter Neely A. (2014). Executive process training in young and old adults. *Aging, Neuropsychology, and Cognition, 21*(5), 577–605. 10.1080/13825585.2013.839777 10.1080/13825585.2013.839777 24148093

[CR85] Shatil, E. (2013). Does combined cognitive training and physical activity training enhance cognitive abilities more than either alone? A four-condition randomized controlled trial among healthy older adults. *Frontiers in aging neuroscience, 26*(5), 8. 10.3389/fnagi.2013.00008 10.3389/fnagi.2013.00008 PMC3607803 23531885

[CR86] Shatil, E., Mikulecká, J., Bellotti, F., & Bureš, V. (2014). Novel television-based cognitive training improves working memory and executive function. *PLoS One, 3*, 9(7), e101472. 10.1371/journal.pone.0101472 10.1371/journal.pone.0101472 PMC4081563 24992187

[CR87] Simon, S., Tusch, E., Håkansson, K., Mohammed, A., & Daffner, K. (2017). Cognitive Changes after Working Memory Training in Healthy Older Adults: Evidence From a Multi-Site, Randomized Controlled Trial. *Neurology, 88*, (16 Supplement) P6.107. 10.3233/JAD-180455 30103334

[CR88] Simon SS, Tusch ES, Feng NC, Håkansson K, Mohammed AH, Daffner KR (2018). Is Computerized Working Memory Training Effective in Healthy Older Adults? Evidence from a Multi-Site, Randomized Controlled Trial. Journal of Alzheimer's Disease.

[CR89] Simpson T, Camfield D, Pipingas A, Macpherson H, Stough C (2012). Improved Processing Speed: Online Computer-based Cognitive Training in Older Adults. Educational Gerontology.

[CR90] Smith GE, Housen P, Yaffe K, Ruff R, Kennison RF, Mahncke HW (2009). A cognitive training program based on principles of brain plasticity: Results from the Improvement in Memory with Plasticity-based Adaptive Cognitive Training (IMPACT) study. Journal of the American Geriatrics Society.

[CR91] Souders, D. J., Boot, W. R., Blocker, K., Vitale, T., Roque, N. A, & Charness, N. (2017). Evidence for Narrow Transfer after Short-Term Cognitive Training in Older Adults. *Frontiers in Aging Neuroscience, 28*(9), 41. 10.3389/fnagi.2017.00041 10.3389/fnagi.2017.00041 PMC5328998 28293188

[CR92] Sosa GW, Lagana L (2019). The effects of video game training on the cognitive functioning of older adults: A community-based randomized controlled trial. Archives of Gerontology and Geriatrics.

[CR93] Stern, Y., Blumen, H. M., Rich, L. W., Richards, A., Herzberg, G., & Gopher, D. (2011). Space Fortress game training and executive control in older adults: a pilot intervention. *Aging, Neuropsychology, and Cognition, 18*(6), 653–77. 10.1080/13825585.2011.613450 10.1080/13825585.2011.613450 PMC3591462 21988726

[CR94] Sterne JAC, Savović J, Page MJ, Elbers RG, Blencowe NS, Boutron I (2019). RoB 2: A revised tool for assessing risk of bias in randomised trials. BMJ.

[CR95] Ten Brinke L. F., Best, J. R., Chan, J. L. C., Ghag, C., Erickson, K. I., Handy, T. C., et al. (2020). The Effects of Computerized Cognitive Training With and Without Physical Exercise on Cognitive Function in Older Adults: An 8-Week Randomized Controlled Trial. *The Journals of Gerontology: Series A, 75*(4), 755–763. 10.1093/gerona/glz115 10.1093/gerona/glz115 31054254

[CR96] Toril P, Reales JM, Mayas J, Ballesteros S (2016). Video Game Training Enhances Visuospatial Working Memory and Episodic Memory in Older Adults. Frontiers in Human Neuroscience.

[CR97] van het Reve E, de Bruin ED (2014). Strength-balance supplemented with computerized cognitive training to improve dual task gait and divided attention in older adults: A multicenter randomized-controlled trial. BMC Geriatrics.

[CR98] van Muijden J, Band GP, Hommel B (2012). Online games training aging brains: Limited transfer to cognitive control functions. Frontiers in Human Neuroscience.

[CR99] Van Vleet TM, DeGutis JM, Merzenich MM, Simpson GV, Zomet A, Dabit S (2016). Targeting alertness to improve cognition in older adults: A preliminary report of benefits in executive function and skill acquisition. Cortex.

[CR100] Vance D, Dawson J, Wadley V, Edwards J, Roenker D, Rizzo M (2007). The accelerate study: The longitudinal effect of speed of processing training on cognitive performance of older adults. Rehabilitation Psychology.

[CR101] von Bastian CC, Langer N, Jancke L, Oberauer K (2013). Effects of working memory training in young and old adults. Memory and Cognition.

[CR102] Wang M-Y, Chang C-Y, Su S-Y (2011). What’s Cooking? – Cognitive Training of Executive Function in the Elderly. Frontiers in Psychology.

[CR103] Wayne, R. V., Hamilton, C., Jones Huyck, J., & Johnsrude, I. S. (2016). Working Memory Training and Speech in Noise Comprehension in Older Adults. *Frontiers in Aging Neuroscience, 22*(8), 49. 10.3389/fnagi.2016.00049 10.3389/fnagi.2016.00049 PMC4801856 27047370

[CR104] Weicker J, Hudl N, Frisch S, Lepsien J, Mueller K, Villringer A (2018). WOME: Theory-Based Working Memory Training - A Placebo-Controlled, Double-Blind Evaluation in Older Adults. Frontiers in Aging Neuroscience.

[CR105] West RK, Rabin LA, Silverman JM, Moshier E, Sano M, Beeri MS (2020). Short-term computerized cognitive training does not improve cognition compared to an active control in non-demented adults aged 80 years and above. International Psychogeriatrics.

[CR106] Wolinsky FD, Vander Weg MW, Howren MB, Jones MP, Martin R, Luger TM (2011). Interim analyses from a randomised controlled trial to improve visual processing speed in older adults: The Iowa Healthy and Active Minds Study. British Medical Journal Open.

[CR107] Zimmermann, K., von Bastian, C. C., Röcke, C., Martin, M., & Eschen, A. (2016). Transfer after process-based object-location memory training in healthy older adults. *Psychology and Aging, 31*(7), 798–814. 10.1037/pag0000123 10.1037/pag0000123 27831716

