# Supplementary material for: Behaviour Change Techniques in Computerized Cognitive Training for Cognitively Healthy Older Adults: A Systematic Review
Source: Neuropsychol Rev. 2022 Feb 14;33(1):238–54. doi: 10.1007/s11065-022-09537-4 (PMC9998598; doi:10.1007/s11065-022-09537-4)
Supplement: Supplementary file 1 — Supplementary file1 (DOCX 18 KB) [file 11065_2022_9537_MOESM1_ESM.docx]

Supplemental File 1: Examples of how Behaviour Change Techniques were applied in the studies.

| **BCTs** | **Examples** |
| --- | --- |
| **Non-Specific Reward** | **Kuhn et al. (2017):**   - To make the game more appealing to an older population, it was set on a recreational cruise ship and called “Schiff Ahoi!” (ship ahoy!). Participants embarked on a virtual cruise of the Mediterranean Sea and could make their ship travel forward on a nautical chart, depending on the amount of points they collected. Throughout their journey, participants arrived at eight different cities along the Mediterranean coastline (e.g. Livorno, Rome, Monte Carlo) and upon arrival at each of these cities they were rewarded with a special virtual postcard. |
| **Feedback on behaviour - by a Computer** | **Payne (2017):**   - Once participants made a decision, the target word would disappear and participants would be presented with accuracy feedback (a green check mark if correct, or a red cross if incorrect) for 1 s. |
| **Monitoring of outcomes of behaviour without feedback - By a Person** | **Toril et al. (2016):**   - Participants assigned to the experimental group completed 15 1-h training sessions at the community senior center in the presence of the experimenter over a period of 7–8 weeks |
| **Feedback on Outcomes of Behaviour - by a Person** | **Fraser et al. (2016):**   - Participants were provided continuous feedback on their response time (during the session) and provided feedback for both response time and accuracy at the end of each session.   **Bozoki et al. (2013):**   - Participant activity time was monitored weekly, and a trouble-shooting/encouraging phone call was initiated by a study staff member if less than 1 h of total activity time was logged for that week. |
| **Self-monitoring of outcomes of behaviour** | **Ackerman et al. (2010):**   - On each day, they were told to complete at least one practice session for each of the 15 mini-games on the “Medium” difficulty setting and record their scores (a level that pretesting indicated would be challenging throughout the practice sessions, and not result in ceiling effects). Additionally, participants were to take one complete test each day and record their overall score and letter grade for that day. |
| **Credible Source** | **Maillot et al. (2012):**   - Each training session was supervised by a physical trainer. The coach’s functions were (a) to implement the training schedule and give any necessary explanations, (b) to ensure the safety of the participants and particularly to prevent the risk of fall, (c) to prevent participants from using incorrect postures or movements or cheating movements, and (d) to give participants feedback on their performance and note their exercise adherence. The participants were urged to try to increase the level of challenge and to improve their performance on each activity over the course of the training |
| **Monitoring of outcomes of behaviour without feedback - by a Computer** | **Ballesteros et al. (2014):**   - Participants practiced the games in our laboratory on a PC equipped with a 21-inch monitor. Scores on each game were recorded. |
| **Self-monitoring of behaviour** | **Hynes et al. (2016):**   - Participants were also encouraged to keep logs and set goals. Participants were given log books where they could track their progress, set everyday and training goals and a summary was provided of each of the training videos. |
| **Graded Tasks** | **Eggenberger (2015):**   - Training difficulty was adapted to each individual’s coordination ability and was increased progressively. |
| **Monitoring of behaviour by others without feedback** | **Akimoto et al. (2016):**   - Group V trained in a vehicle with an on-board cognitive training program. In the training task, color stimuli were presented rhythmically and randomly by five LEDs placed around the driver’s seat (four lights: top-right, bottom-right, top-left, and bottom-left were approximately equidistant from the center of the driver’s view; an additional LED was located beside the side mirror on the passenger side). Participants were instructed to turn the steering wheel when two lights of the same color (green or blue, but not yellow) were presented on the same side (left or right). Yellow lights were distractors, so participants were expected to suppress their response even if two yellow lights were presented on the same side. Participants were also instructed to press the brake pedal when red lights were presented at random times by any of the five LEDs. |
| **Monitoring of behaviour by others without feedback – by a computer** | **Zimmerman et al. (2016):**   - Training was self-administered at home using the open-source Java-based software Tatool (von Bastian, Locher, & Ruflin, 2013; www.tatool.ch). After each training session, data were automatically uploaded to a web server, allowing for constant monitoring of participants’ compliance. |
| **Demonstration of the behaviour** | **Souders et al. (2017):**   - Participants then attended a 2-h training session, which involved a tutorial on how to use the provided tablet (10 inch Acer Iconia A700), as well as how to play the games they were assigned per their condition.   **Buitenweg et al. (2017):**   - A separate test assessor administered four computer tasks, and introduced the training to subjects using instruction videos and a demonstration of the training platform and games. |
| **Verbal persuasion about capability** | **Guye et al. (2017):**   - To enhance training commitment, participants were individually reminded via e-mail if they fell behind their training schedule. Moreover, at the beginning of every training week, participants received an e-mail with information on their training status and a motivating slogan (e.g., “If you always do what you’ve always done, you’ll always get what you’ve always got”). |

BCTs were only included in this list if they were associated with either:

- A statistically significant difference in efficacy (p<0.05, Table 2)
- A clinically relevant difference in efficacy (difference in Hedges’ g <-0.10 or >0.10, Table 2)
- A clinical significant difference in adherence (p<0.05, Table 4)
